# Sphingomyelin in High-Density Lipoproteins: Structural Role and Biological Function

**DOI:** 10.3390/ijms14047716

**Published:** 2013-04-09

**Authors:** Roberto Martínez-Beamonte, Jose M. Lou-Bonafonte, María V. Martínez-Gracia, Jesús Osada

**Affiliations:** 1 Departamento de Bioquímica y Biología Molecular y Celular, Facultad de Veterinaria, Instituto de Investigación Sanitaria de Aragón-Universidad de Zaragoza, Zaragoza E-50013, Spain; E-Mail: romartin@unizar.es; 2 CIBER de Fisiopatología de la Obesidad y Nutrición, Instituto de Salud Carlos III, Madrid E-28029, Spain; E-Mails: mlou@unizar.es (J.M.L.-B.); mvmartig@unizar.es (M.V.M.-G.); 3 Departamento de Farmacología y Fisiología, Facultad de Ciencias de la Salud y del Deporte, Universidad de Zaragoza, Huesca E-22002, Spain

**Keywords:** high-density lipoproteins, phospholipids, sphingomyelin

## Abstract

High-density lipoprotein (HDL) levels are an inverse risk factor for cardiovascular diseases, and sphingomyelin (SM) is the second most abundant phospholipid component and the major sphingolipid in HDL. Considering the marked presence of SM, the present review has focused on the current knowledge about this phospholipid by addressing its variable distribution among HDL lipoparticles, how they acquire this phospholipid, and the important role that SM plays in regulating their fluidity and cholesterol efflux from different cells. In addition, plasma enzymes involved in HDL metabolism such as lecithin–cholesterol acyltransferase or phospholipid transfer protein are inhibited by HDL SM content. Likewise, HDL SM levels are influenced by dietary maneuvers (source of protein or fat), drugs (statins or diuretics) and modified in diseases such as diabetes, renal failure or Niemann–Pick disease. Furthermore, increased levels of HDL SM have been shown to be an inverse risk factor for coronary heart disease. The complexity of SM species, described using new lipidomic methodologies, and their distribution in different HDL particles under many experimental conditions are promising avenues for further research in the future.

## 1. Introduction

The high-density lipoproteins (HDL) are the smallest and densest of the plasma lipoproteins and are approximately one half protein and one half lipid by weight. The principal protein constituent of HDL is apolipoprotein A1 (APOA1) followed by APOA2; minor protein components are APOA4, APOD, APOE, APOF, APOH, APOJ and APOM. HDL also transport enzymes such as lecithin–cholesterol acyltransferase (LCAT), paraoxonase 1 and 3 and platelet-activating factor acetylhydrolase, and proteins involved in regulating the complement system and protecting tissue from proteolysis [1]. The nascent HDL are secreted by the gut and liver. A current model of nascent or newly secreted HDL proposes that they are formed by a bilayer of phospholipids surrounded by four apolipoprotein molecules. Considering the number of apolipoproteins and the limitation per particle, the range of particles is wide (Table 1) [2]. These particles incorporate cholesteryl esters into their interior, forming their hydrophobic core through the action of LCAT, which transfers a fatty acid moiety from phosphatidylcholine (PC) to cholesterol and forms cholesteryl ester. The incorporation of this compound facilitates the formation of a stable spherical particle, depending on the size, referred to as HDL_2_ or HDL_3_. The former are larger than HDL_3_ and contain more lipid-rich particles, whereas HDL_3_ are relatively protein-rich, lipid-poor and dense [3]. Another source of nascent HDL is hydrolysis of the triglyceride-rich lipoprotein particles-chylomicrons and very-low-density lipoproteins which results in the formation of HDL_2_. Different states of maturation also result in a wide range of HDL sizes—26, according to nuclear magnetic resonance technology [4] (Table 1).

A low level of high-density lipoprotein cholesterol (HDL-C) is a powerful risk factor for cardiovascular disease. The exact role of HDL in atheroprotection is not yet well understood, in part due to the existence of different lipoparticles (Table 1) that vary in size, composition and function [2]. Furthermore, oxidative modification of these particles at the lipid [6,7] or protein level [8] increases the heterogeneity and renders them dysfunctional and pro-atherosclerotic [9]. Isolation and deep characterization of these lipoparticles will provide crucial information for understanding the function of HDL in health and disease.

The major lipid constituents of HDL are phospholipids-PC, SM and lysoPC, followed by cholesterol and cholesteryl ester. HDL also carries other lipids including sterols, triglycerides, fat-soluble vitamins, ceramide, sphingosine-1-phosphate and dihydrosphingosine-1-phosphate [10] (Recent reviews addressing their function can be found in [11,12]). New analytical technologies to tackle the HDL lipidome are characterizing hundreds of lipid compounds based on the fatty acids present in these compounds [10,12,13] and their physiological and pathological variations await further research.

**Figure 1:**
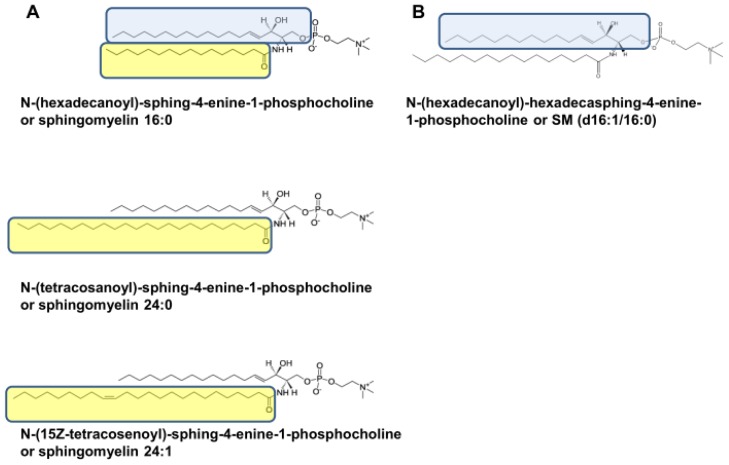
Different molecules of sphingomyelin according to the length and unsaturation of the acyl residue and sphingoid. A, fatty acid compositions of sphingomyelin species (yellow box) and A and B, long-chain base compositions of sphingomyelins (blue box). Note that the major difference among SM molecules is length and saturation of fatty acids. (http://www.lipidmaps.org/data/structure/LMSDSearch.php?Mode=ProcessClassSearch&LMID=LMSP0301&s=sphingomyelin).

**Figure 2:**
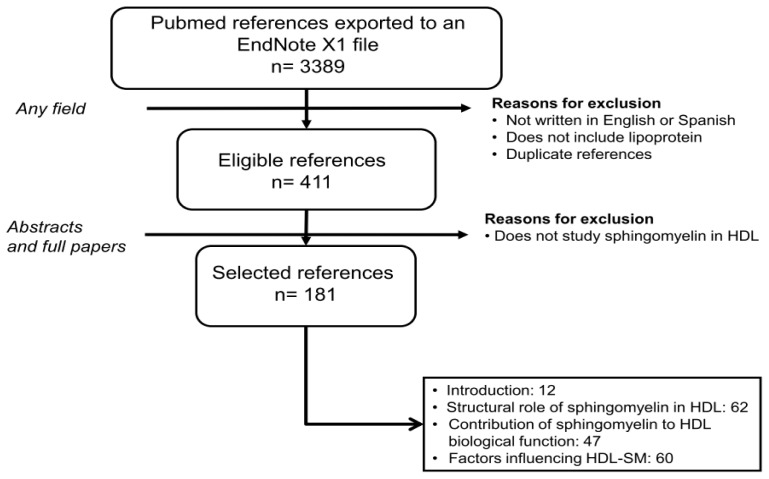
Flow chart displaying the stages used to select the references considered. The present report has adhered to systematic review guidelines [14]. A search in Pubmed (http://www.ncbi.nlm.nih.gov/pubmed/) using the keywords “high-density lipoprotein and sphingomyelin,” “sphingomyelin and diet,” “sphingomyelin and drugs,” “plasma and sphingomyelin,” identified 3,389 hits from November 1945 to March 2013. Documents that failed to meet the criteria shown were discarded. Thus, this review covers the works related to sphingomyelin and HDL in 181 papers. EndNote X1 (Bld 2566, Thomson Reuters: New York, NY, USA, 2007).

Sphingomyelin or *N*-acyl-sphing-4-enine-1-phosphocholine encompasses a wide range of compounds depending on the length and unsaturation of the acyl residue and the length of the sphingoid bases (Figure 1), the former being more relevant in terms of structural changes. Despite the fact that SM is the second most abundant phospholipid and the main sphingolipid in HDL [10], its physiological function is unclear. To make advancements in the field, the present review has been carried out according to the criteria shown in Figure 2.

## 2. Structural Role of Sphingomyelin in HDL

### 2.1. Distribution into HDL Subclasses

As previously mentioned, SM is the second most abundant phospholipid in HDL. However, there is a wide heterogeneity among HDL particles. Indeed, SM decreased in abundance in parallel with the progressive increase in HDL density from HDL_2_ (19%) to HDL_3_ (6.5%) [5,10,15–17]. Other authors have found higher proportions of SM for the latter particle. Davidson *et al.* reported that the HDL_3_ phospholipid fraction in plasma from five normolipidemic subjects contained 12% SM [18]. This discrepancy could be explained by the inhibition of phospholipid transfer protein or hepatic lipase during the isolation procedure, as referred to by Marques-Vidal *et al.*[19], or the moment of extraction, since fasting is an important factor [10]. The distribution of phospholipids in HDL is similar in other species, although SM content may vary widely from 4.5% in mouse to 19.7% in American armadillo [20]. Intermediate values are observed in baboon (5.9%) [21], dog (8.7%) and cat (10%) [20]. In all of them, SM represents the second most abundant phospholipid and this phylogenetic conservation may suggest an important role.

Heterogeneity among subclasses of HDL with respect to content of SM has also been observed using immunoaffinity chromatography. In this regard, HDL_3_ containing APOD were found to be particularly rich in this phospholipid [22], as were gamma-LpE, an HDL containing APOE [23], and those containing APOA4 or APOA1/APOA2/APOA4 [24]. However, using filtration through a polyacrylamide gel under the effect of an electric field, the seven HDL subclasses observed, with respective molecular masses of 42–50, 71, 103, 124, 150, 182 and 219 kDa, showed a similar composition [25]. The source of HDL also generates variation. In fact, lymph HDL lipids had a significantly higher SM content expressed in terms of its relationship to PC (SM/PC ratio) [26]. Lymph HDL apoprotein composition differs from that of plasma, with an increase in APOE and APOA4 content relative to APOA1. These discoidal HDL particles could be products of an initial stage of reverse cholesterol transport [27].

### 2.2. Source of Sphingomyelin

The SM for lipoprotein is formed through several reactions: the first one, catalyzed by serine–palmitoyl transferase, forms dihydrosphingosine from palmitic acid and serine. Dihydrosphingosine is converted into ceramide in three sequential steps catalyzed by dihydrosphingosine reductase, dihydroceramide synthase and dihydroceramide reductase. Sphingomyelin synthase (SMS) catalyzes the transfer of phosphocholine moiety from PC to ceramide, resulting in SM and diacylglycerol [28]. Two enzymes, SMS1 located at the Golgi and SMS2 at the plasma membrane, have been characterized [29], and SM can be added to APOA1 at both locations [30]. In the plasma membrane, SMS2 is located in membrane domains rich in SM and cholesterol referred to as rafts [29,31]. Besides the original phospholipids contained in nascent HDL, the efflux of these lipids from cells contributes to the phospholipid content of HDL. In this regard, HDL_3_ promoted the efflux of phospholipids from human skin fibroblasts in a concentration-dependent manner and the major phospholipids released were PC, SM, lysoPC and phosphatidylethanolamine [32]. Lipid-free APOA1, interacting with specific regions of plasma membrane fibroblasts, is able to acquire phospholipids [33]. Based on the similarity of lipid composition of nascent HDL and lipid rafts, it has been proposed that these raft-like regions may provide the lipids to HDL [34]. Using fibroblasts from Niemann–Pick type C patients, APOA1-mediated efflux of PC and SM was reduced, suggesting that the cholesterol trafficking defect observed in this disease influences HDL characteristics [35]. APOA1 binding to artery-derived human smooth muscle cells depleted them of PC and SM. In contrast, rat smooth muscle cells or human vein-derived cells released only a small fraction of these phospholipids to APOA1-containing medium. In Raw 264.7 macrophages and CHOK1 cells, only PC (but not SM) was found to be effluxed from both cell lines [36]. The marked difference between these cells may reflect cell- and species-specific mechanisms of phospholipid efflux [37,38]. Similar differences among species were found for phospholipid transfer protein regarding selective transfer of SM between membrane surfaces [39]. It has also been noted that the use of APOA1 or HDL_3_ makes a difference with respect to the phospholipid species effluxed from fibroblasts and macrophages. In this regard, medium chain SM species containing 14:0 or 16:1 were translocated preferentially to APOA1 and not to HDL_3_[40].

### 2.3. Interaction of SM with Apolipoproteins

Several experiments have been undertaken to understand this aspect. SM accelerated the formation of reconstituted HDL by APOA1 when added to unilamellar vesicles comprised of PC [41]. In addition, it modified HDL particle size and stability and the negative surface charge of APOA1 [42]. Treatment of the HDL_3_ with sphingomyelinase induced a progressive fluidization which suggests that SM together with PC modulates the fluidity and order of the surface of HDL_3_[43,44] and the SM/PC ratio is considered to be an index of rigidity. Moreover, complete degradation of SM resulted in an increase in the rate of HDL_3_ cholesterol oxidation [45], suggesting an increased exposure of the compound or an antioxidant action of SM, as reported in other lipoproteins [46].

**Figure 3:**
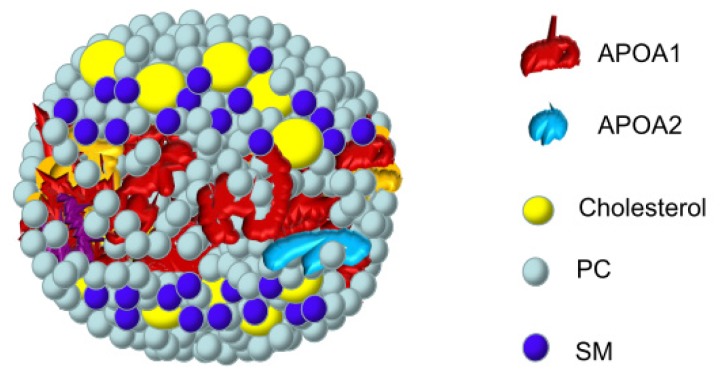
Location of SM and other surface components of HDL. Phosphate choline groups of sphingomyelin on the surface are depicted close to cholesterol molecules in a HDL lipoparticle.

In HDL, SM was located in a hydrophilic environment and its interaction with apolipoproteins was not particularly relevant in the formation of these plasma complexes (Figure 3) [47,48]. In fact, it was proposed that the stability of the vesicle lipoprotein was related to the cholesterol/phospholipid ratio in these particles [49,50]. Compared with SM liposomes, the polar head groups of these lipids in the lipoprotein particles displayed a considerably higher mobility [51]. Not surprisingly, the molecular interactions and spatial arrangements of phospholipids and apolipoproteins of human HDL revealed a spatial relationship between the hydrophobic side chains of the lipid and polypeptide chains in the lipoprotein complex [52]. The mechanisms of interaction between APOA1 and either bovine brain SM or PC are similar, but the nature of the protein–lipid interactions with SM allows the production of larger and more stable complexes than those observed with PC [53]. While a more extensive interaction was described for APOA1, SM interacts with the COOH– but not the NH_2_-terminal region of APOA2 [54]. For this reason, APOA1 recruited significantly more phospholipid and cholesterol than APOA2 and showed a special preference for SM [55–57]. Using labeled SM, it was possible to demonstrate a differential interaction with the apoprotein tryptophans, as well as a non-random distribution at the surface of the particles [58]. In APOA1 Seattle (deletion of amino acids Glu146 to Arg160), nascent HDL showed a significant increase in SM compared to wild type nascent HDL [59], suggesting a better availability of interacting regions.

### 2.4. Different SM Species

The existence of different SM molecules has been mentioned earlier (Figure 1). In this respect, a peculiarity of SM from HDL in normolipemic subjects is that their molecular weights are higher than those of other lipoproteins due to the longer chain length of the fatty acids in the ceramide molecules. Indeed, SM 24:1 was the second most abundant species both in HDL_2_ and HDL_3_[10], but its plasma levels decreased in response to diabetes induction and high-fat diets [60], and SM 23:3 acted as a discriminator among subjects with different HDL levels [61]. This has been interpreted as a lateral phase separation of the short and long-chain SM during the shedding of the excess VLDL or chylomicron surface material and a subsequent preferential transformation of the long-chain species into HDL [62–65]. Using liquid chromatography-mass spectrometry, SM 16:0 has been found to be the predominant species in human HDL [10,66], although it is present in a much smaller amount than in LDL [67]. Sphingomyelin 16:0 was found to be more elevated in HDL_2_ than HDL_3_[10]. This differential composition seems to develop in adults since, in suckling rats, the fatty acid composition of SM was relatively constant in all lipoprotein fractions [68].

### 2.5. Metabolic Fate of HDL Sphingomyelin

The effects of the removal of SM from HDL have been examined in several tissues. While the rat brain hardly utilizes very long-chain fatty acids from HDL SM, even during the myelination period [69], many other tissues may take up intact SM from HDL and hydrolyze it [70]. Treatment of HDL with hepatic lipase did not affect the delivery of SM [71]. In contrast, pretreating platelets with elastase dose-dependently inhibited uptake of SM from HDL. Therefore, the uptake of this phospholipid appears to require the presence of specialized platelet membrane proteins [72]. These findings indicate a selective mechanism for removal of SM from tissue that needs to be characterized molecularly.

*In vivo* transfer of SM from HDL to VLDL was also observed [73,74]. The same occurred between LDL and HDL, and inhibition of plasma LCAT reduced the exchange of SM [75].

## 3. Contribution of Sphingomyelin to the Biological Function of HDL

The following sections cover the main actions of HDL in which SM has been shown to participate.

### 3.1. Reverse Cholesterol Transport

**Figure 4:**
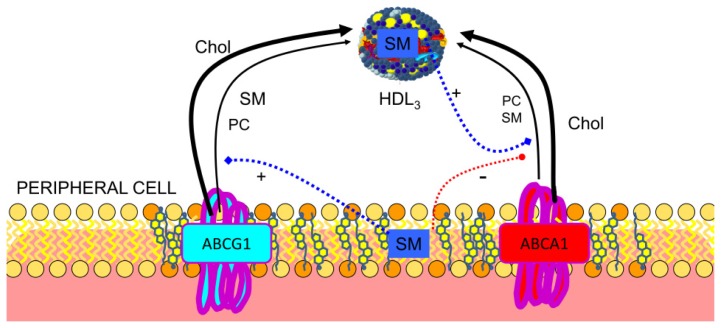
Current model of SM on cholesterol efflux. SM both in HDL and cell membrane regulates ATP binding cassette proteins implicated in reverse cholesterol transport. Cellular SM has been described as a stimulant of SM and cholesterol efflux through ABCG1, ATP-binding cassette transporter G1 [92]. On the other hand, cell membrane SM inhibits ABCA1, ATP-binding cassette transporter A1 activity [102], while SM present in HDL stimulates activity of this transporter [103].

The inverse association between HDL cholesterol and coronary heart disease has been attributed to reverse cholesterol transport, this being the main function of HDL, with cellular efflux as the first step in the process. The proportion of HDL-SM could be used to predict the capacity of serum to accept cellular cholesterol since it was positively correlated with its fractional efflux [76]. Moreover, HDL-SM had the strongest inverse association with the presence of coronary heart disease among all HDL-related parameters upon multivariate analysis of data from women with angiographically assessed disease. In fact, it was the only HDL-related parameter that had a significant and independent correlation with the number of coronary stenoses [77,78]. Early experiments of Stein *et al.* demonstrated that the removal of cholesterol could be enhanced by addition of sonicated suspensions of PC or SM to human high-density apolipoproteins [79–82]. Using reconstituted discoidal HDL particles prepared with APOA1, it was shown that increasing the content of SM, up to 20 mol/particle, was associated with significantly increased abilities of the HDL to promote cholesterol efflux from non-cholesterol-loaded human skin fibroblasts [83], from erythrocyte ghost membranes [84] or from Fu5AH cells [85]. Sphingomyelin promoted similar cholesterol efflux in control, familial HDL deficiency or Tangier disease fibroblasts [86]. It was concluded that discoidal, phospholipid-rich recombinant lipoproteins could effectively take up substantial amounts of cholesterol from physiological membranes, provided that the phospholipids utilized to form micellar complexes retained their structural integrity during the incubation. Reconstituted HDL with sphingomyelin and APOA1 Milano promoted the greatest cholesterol efflux in each cell type (CHO cells, J774 macrophages, and BHK cells) and this was enhanced by increased expression of ATP-binding cassette sub-family G member 1 **(**ABCG1) [28,87]. This transporter has been shown to preferentially secrete SM [88,89], in contrast to ABCA1 [90], and this phospholipid stimulated the ATPase activity of ABCG1 and increased the affinity for cholesterol, suggesting different binding sites for cholesterol and SM, which may be synergistically coupled [91]. On the other hand, the ABCG1-mediated efflux of cholesterol and SM is dependent on the cellular SM level and distribution of cholesterol in the plasma membrane [92], as reflected in Figure 4. Furthermore, HDL-mediated cholesterol efflux was partially inhibited by sphingomyelinase treatment [93]. The situation may be more complex since high SM content decreased uptake in reconstituted HDL-containing linolenic acid, whereas it increased efflux for reconstituted HDL-containing oleic or linoleic acid [94]. Other authors have confirmed these findings [95] and suggest that HDL removes cholesterol depending on HDL fatty acids. However, increasing membrane SM content increased the time required for cholesterol exchange in both the erythrocyte plasma membrane and in simple mixed SM/PC bilayers [96]. When Slotte *et al.* used HDL_3_ as a physiological acceptor for cholesterol, treatment of cells with sphingomyelinase induced a dramatically increased esterification of plasma-membrane-derived [^3^H]-cholesterol [97]. An increase in the level of [^3^H]-cholesterol efflux to HDL was also observed after degradation of plasma membrane SM with exogenous sphingomyelinase in fibroblast plasma membranes [98,99]. Under these conditions, cellular release of cholesterol may be enhanced by a ceramide-signaling cascade after sphingomyelinase treatment [100] or SM depletion [101]. These results show the importance of SM in reverse cholesterol transport and that cholesterol molecules interacting with SM in the plasma membrane domain appear to be primarily used for HDL assembly when they interact with cells. Thus, due to its specific interactions with cholesterol, SM may represent an important factor accounting for the inverse association between HDL cholesterol and coronary heart disease.

### 3.2. Regulation of ABCA1

ATP-binding cassette protein A1 (ABCA1) plays a major role in cholesterol homeostasis and HDL metabolism, specifically its activity is critical for HDL biogenesis. APOA1 binds to ABCA1 and this translocates phospholipids and cholesterol directly or indirectly to form pre-β HDL. Downregulation of ABCA1 expression by interferon–gamma in murine peritoneal macrophages resulted in reduced PC and SM efflux to APOA1 [104]. It has been suggested that the ATPase activity of ABCA1 is stimulated preferentially by PC and SM [103] and that ABCA1 stability is controlled by apolipoprotein-mediated signaling requiring SM since digestion of this lipid increased ABCA1 stability [102]. Nevertheless, Tamehiro *et al.* demonstrated that serine palmitoyltransferase enzyme subunit 1 negatively regulated ABCA1 function by a physical interaction and that a reduced level of sphingomyelin synthesis was not involved [105].

### 3.3. Regulation of Plasma Enzymes

**Figure 5:**
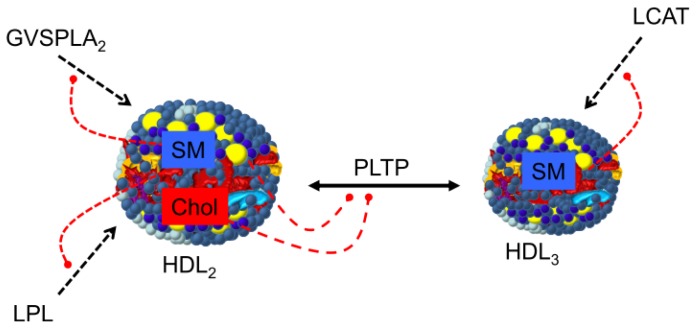
Role of HDL-SM in modulating enzymes of HDL metabolism. SM and cholesterol have been described as inhibitors of PLTP, phospholipid transfer protein. SM has been also described as inhibitor of GVSPLA_2_, group V secretory phospholipase A_2_, LPL, lipoprotein lipase and LCAT enzymes [42,107–111].

The activity of several enzymes (phospholipid transfer protein group, group V secretory phospholipase A_2_, lipoprotein lipase and LCAT) has been found to be modulated by SM, as reflected in Figure 5. Early experiments showed that SM played a role in phospholipid transfer from liposomes to human HDL. In fact, an increase in the amount of bovine brain SM in egg PC liposomes decreased phospholipid leakage from said liposomes in the presence of serum. Sphingomyelin itself was not readily exchanged with HDL, possibly due to intermolecular hydrogen bonding between the sphingosine backbones of the SM molecule [106]. Sweeney and Jonas later proved that the activity was attributable to human plasma phospholipid transfer protein and that this was inhibited by SM, as well [107].

Group V secretory phospholipase A_2_ activity was also inhibited by SM [112] and, in addition, SM modifies selectivity for arachidonate [108,113]. Likewise, increasing the amount of SM (up to 22 mol%) in reconstituted HDL-containing PC, cholesterol and APOA1 was found to inhibit lipoprotein lipase [114].

Using reconstituted HDL, it was found that LCAT was inhibited by up to 90% in the presence of SM, an effect that was less pronounced when accumulation of cholesteryl ester product directly inhibited LCAT [115], and reversed by treatment with bacterial sphingomyelinase. Kinetic studies showed that LCAT binds more effectively to vesicles with SM, and that the latter competes with the substrate PC [42,43,109,116]. A relationship between plasma SM content and LCAT activity has been shown in several animal models, reinforcing *in vitro* data and pointing to SM as a physiological modulator of LCAT activity [110,117].

### 3.4. SR-BI in Lipid Transfer to the Cells

**Figure 6:**
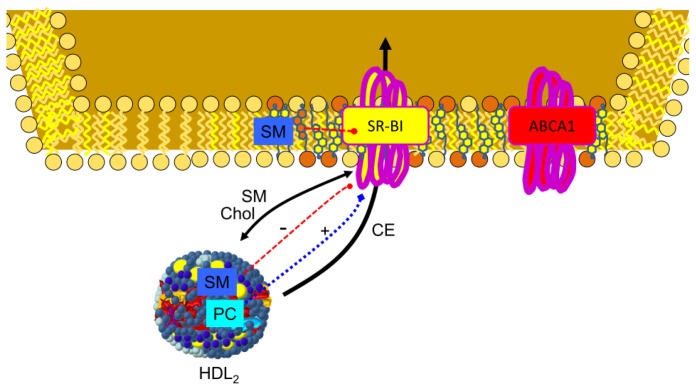
Influence of SM in delivering cholesterol to cells through the SR-BI receptor. SR-BI mediates cholesterol ester influx by the cells but also bidirectional flux of unesterified cholesterol and phospholipids between HDL and cells. Both cell membrane and HDL SM inhibit SR-BI transport activity, while HDL PC stimulates its transport activity [31,121,122].

Scavenger receptor BI (SR-BI) is the first molecularly defined receptor for HDL. It can mediate the selective uptake of cholesteryl ester into cells with no or partial SM uptake. This requires integrity of membrane rafts and occurs via a retroendocytic pathway in HepG2 cells [118,119]. The interaction of APOA1 with SR-BI not only prevents fusion of the lipid donor with the plasma membrane, but also allows the optimal selective lipid uptake [120]. The role of HDL phospholipid in SR-BI-mediated free cholesterol flux was examined by manipulating HDL_3_ PC and SM content, and it was found that enrichment of HDL with either of these phospholipids enhanced the net efflux of cholesterol from SR-BI-expressing COS-7 cells, but by two different mechanisms. Phosphatidylcholine enrichment of HDL increased efflux, whereas SM enrichment decreased influx of HDL cholesterol [121]. Subbaiah *et al.* confirmed that incorporation of SM into reconstituted HDL strongly inhibited the uptake of cholesterol esters and that this inhibition was completely reversed by treatment of HDL with sphingomyelinase [31]. A similar phenomenon was observed using intestinal brush border membrane vesicles and Caco-2 cells in a process mediated by SR-BI and CD36 [122]. Overall, these data indicate that SR-BI-mediated free cholesterol flux is dependent on the structure of the donor lipoprotein [123] and that SM is playing an important role, as reflected in Figure 6.

## 4. Factors Influencing HDL-SM

### 4.1. Aging and Sex

Aging has been proposed to attenuate cholesterol efflux (see review [124]). Among many changes, the composition and structure of HDL revealed an increase in the SM/PC ratio and in the phospholipid membrane fluidity in HDL in the elderly compared with HDL in younger individuals, as well as an alteration in the APOA1 structure and charge. Interestingly, reduction in the HDL-mediated cholesterol efflux capacity with aging was more significant with HDL_3_ than HDL_2_. ABCA1-mediated cholesterol efflux was the more affected pathway in terms of cholesterol-removing capacity [125].

The only manuscript studying sex differences showed lower HDL SM values in men than in women. HDL SM values were independent of age in both sexes, with the exception that HDL SM concentrations were lower in men aged 55 years or over [126].

### 4.2. Lifestyle

More prolific is the aspect of dietary influence in terms of species and food components. In *Ldlr*-deficient mice, a diet rich in saturated fats and cholesterol increased HDL-SM, expressed either as nanomole per milliliter of serum or nanomole per milligram of protein [127]. In rats, the source of polysaccharide and protein and the nature of fat influence HDL SM. For example, pectin (20 g/100 g) increased SM content in the HDL_2_ fraction in rats [128], while casein feeding (35 g/100 g) lowered HDL_2_ SM concentrations when compared to soybean protein-fed rats. Plasma transfer of SM from HDL_2_ to VLDL could be involved [129]. Dietary olive oil (180 g/kg) reduced HDL SM content compared to coconut fat in rats. The lower value may be explained by a simultaneous elevation of SM catabolism and reduction in its synthesis [130]. However, a postprandial elevation of HDL SM, accompanied by intestinal expression of SM synthase1 mRNA, was observed in rats receiving a bolus of 5 mL of extra virgin olive oil [131]. Likewise, conflicting results have been observed regarding sphingolipid-rich diets: while HDL-lipids were not significantly altered in LDL receptor gene knockout mice [132], other authors have described changes [133]. More studies addressing different experimental animals and regimes are required to clarify the issue.

In humans, consumption of a diet rich in n-3 polyunsaturated fatty acids seemed to increase SM in HDL_2b_ and HDL_3_, and was associated with higher HDL cholesterol levels in plasma [134]. Sphingomyelin composition was not modified by dietary fat in healthy young male participants [135], nor were any changes observed in subjects consuming various isocaloric diets, each containing 30% of the calories as fat [136]. In the latter study, 15.6% of the total calories were provided successively by olive oil, soybean oil, corn oil or milk fats. In a seven-week double-blinded, randomized, controlled, parallel group study with healthy subjects, an increase in SM of long-chain polyunsaturated fatty acids was observed in subjects receiving capsules containing 8 g/d of fish oil (1.6 g/d EPA + DHA), compared to those consuming 8 g/d of high oleic sunflower oil [137]. Minor dietary components may also influence the results since the consumption of high-polyphenol chocolate increased HDL SM [138]. Plasma SM 16:1 was found to be associated with decreased risk of diabetes [139], and plasma levels of SM 24:1 decreased in response to diabetes induction and high-fat diets [60]. Whether these changes also take place in HDL SM needs to be explored.

Few studies have addressed exercise; regarding this aspect, a single exposure to a 100-km run did not modify HDL SM [140].

Ethanol consumption in rats was found to have decreased HDL SM, with a concomitant decrease in cholesterol efflux compared to that of controls. In humans, plasma HDL SM was also decreased in chronically alcoholic individuals with or without liver disease, compared with nondrinkers. Based on these findings, it was concluded that long-term ethanol consumption significantly impaired cholesterol efflux function of HDL by decreasing its SM content [141]. These results are a truly interesting contribution to the search for a substitute for cholesterol efflux assays in large intervention studies.

### 4.3. Pharmacological Agents

Many pharmacological agents have been observed to exert an effect on HDL SM content. In this respect, after intraperitoneal insulin pump implantation, SM concentrations decreased in all lipoprotein fractions. As a result, the SM/PC ratio, an index of the surface rigidity of lipoproteins, decreased [142], a change that was not observed with continuous subcutaneous insulin infusions [143]. Administration of raloxifene, a selective estrogen receptor modulator, to postmenopausal women also decreased HDL_2_ SM [144], an effect that was not potentiated by combined administration of atorvastatin, an inhibitor of hydroxymethylglutaryl-CoA reductase [145], despite its reported SM-reducing properties. Another statin, rosuvastatin, was even more potent than atorvastatin regarding the latter effect [146].

SM-increasing action has also been reported. In this regard, fenofibrate, a hypocholesterolemic agent acting as ligand of the peroxisome proliferator-activated receptor α, increased HDL SM [147–149], as did the administration of the antihypertensive captopril, an angiotensin-converting enzyme inhibitor, and the diuretic hydrochlorothiazide in hypertensive patients [150]. Contraceptives were also found to exert this effect. This change was most profound in women receiving preparations in which progestin was predominant; in contrast, in women receiving preparations with higher estrogen to progestin ratios, the SM/PC ratio actually declined [151,152]. On the other hand, androgens such as tibolone lowered HDL-cholesterol without modifying HDL SM [153]. These compositional changes in the phospholipid content of HDL may influence its capacity to promote cellular cholesterol efflux, as reported previously, and contribute to the atherogenicity of these agents. In fact, inhibition of *de novo* SM biosynthesis by myriocin, an inhibitor of serine palmitoyltransferase, resulted in decreased atherosclerosis in *Apoe*-deficient mice [154] and induction of APOA1 and LCAT activity [111].

Other agents such as ibuprofen, when added to HDL, induced an upfield chemical shift of SM in lipoprotein particles. This could be explained by changes in orientation of the phospholipid head group at the surface of the lipoprotein particles [155].

### 4.4. Changes in HDL-SM Affected by Pathological Conditions

The amount of SM present in HDL may be modified in several pathological circumstances, both in rare monogenic disorders and in common diseases. A threefold increase in the SM/PC ratio was found in plasma HDL in abetalipoproteinemia, with no changes in SM fatty acid composition [156–158]. Niemann–Pick disease types A and B caused by an acid sphingomyelinase deficiency due to mutations in the sphingomyelin phosphodiesterase-1 gene. These patients have been reported to have a severe reduction in plasma HDL cholesterol [159–161] and SM-enriched HDL [162]. This abnormal HDL composition may cause a decrease in LCAT activity and a lack of HDL maturation [163,164], and was a consequence of a reduced cholesterol efflux [165]. In Tangier disease, the relative quantity of long-chain fatty acids (23 or more carbons) in serum SM was about 38% lower than that of control sera [166].

Said changes are not only found in these low prevalence disorders, but in more common ailments, as well. In this regard, decreased content of HDL SM was observed in the acute-phase response [167], in hypoalphaliproteinemia [13] and in patients with triple-vessel coronary artery disease [168]. Decreased concentrations of HDL SM were also observed in diabetic women [169], while they were not observed in diabetic men [170]. A decreased SM level as compared to PC was found in renal patients on long-term maintenance hemodialysis [171]. Surprisingly, in cross-sectional analyses, type 1 diabetic patients with advanced kidney disease had high serum SM [172], and SM emerged as a significant biochemical covariate of urinary albumin excretion [173]. An increased HDL phospholipid concentration has been shown to be a valuable index in patients with multiple myeloma responding to therapy [174] or after periodontal treatment, as HDL phospholipids and the SM/PC ratio also increased in patients with periodontitis [175]. Higher HDL SM was observed in hypertensive patients [176].

Changes in HDL-SM have also been observed in several strains of knockout or transgenic animals. In this respect, increased SM content was observed in *Abca1*-deficient mice and these mice showed decreased phospholipid transfer protein and LCAT activities [177]. Increased HDL SM was also observed in *Apoe*-deficient mice due to reduced catabolic rate and increased production rates [178]. On the other hand, using recombinant adenovirus vectors containing human SM synthase 1 and 2, it was shown that HDL-SM content decreased due to enhanced clearance of these particles [179,180]. Changes in the SM/PC ratio were also observed in *Pltp*-deficient mice in association with a marked reduction in HDL levels [181]. Liver *Sptlc2* deficiency decreased plasma HDL SM levels by 36%, and increased PC levels, thus decreasing the SM/PC ratio compared with controls [182].

## 5. Conclusions

Sphingomyelin present in HDL modifies their properties by altering protein conformation and function, and exerts an effect on other enzymes, receptors, transporters, *etc*., involved in HDL metabolism. Its level is very dynamic and changes under physiological, pharmacological and pathological conditions. The HDL-SM association with cholesterol efflux is a promising field to be confirmed as a new biomarker in coronary heart disease. Furthermore, the existence of different species of SM based on the fatty acid residue on the sphingoid base and the selective presence of SM in some HDL particles open an important field focusing on isolating and characterizing types of SM in these lipoparticles and their relevance in health and disease. Thus, further studies are warranted to establish the value of all these parameters.

## Figures and Tables

**Table 1:** HDL subspecies depending on analytical preparation.

Procedure	Categories							
Ultracentrifugation (Densities in g/mL)	HDL_2b_ (1.063 to 1.090)		HDL_2a_ (1.090 to 1.120)		HDL_3a_ (1.120 to 1.150)		HDL_3b_ (1.150 to 1.180)	HDL_3c_ (1.180 to 1.210)
Electrophoretic mobility	preβ-1	preβ-2	α-1	α-2	α-3	preα-1	preα-2	preα-3c
Nuclear magnetic resonance	26 subtypes							
Immune affinity	Lipoparticles containing: APOA1, APOA2, APOA4, APOD, APOE, APOF, APOH, APOJ, APOM, APOA1-APOA2, APOA1-APOA2-APOA4……							

Results from four common analytical methods revealing the heterogeneity of HDL lipoparticles, and reflected categories from one method do not necessarily correspond to another. Adapted from [4,5].

## Abbreviations

APO: apolipoprotein
CHD: coronary heart disease
CM: chylomicrons
HDL: high-density lipoproteins
LCAT: lecithin–cholesterol acyltransferase
LDL: low-density lipoproteins
PC: phosphatidylcholine
SM: sphingomyelin
VLDL: very low-density lipoprotein
